# Contribution to the Etiology of Neuralgia

**Published:** 1888-05

**Authors:** 


### Contribution to the Etiology of Neuralgia.

In the *Edinburgh Med. Journal*, December, 1887, Mr. James R. Whitehall, says there are many conditions classed under the heading neuralgia. There is the pure form, with no apparent and definite cause during life, and leaving no discoverable lesion after death; the reflex form, which may be, and frequently is, due to dental caries and other remote causes, the removal of which gives speedy relief to the symptoms. Then, again, there are other forms due to an actual inflammation of the nerve—a true neuritis and not a neuralgia at all; others due to poisons circulating in the blood, such as lead, the poison of syphilis, gout and malaria; and some to pressure upon the trunk of the affected nerve, or to an irritation still higher in the course of the nerve. In the pure form of neuralgia, where there is no apparent cause except the debility so frequently associated with it, the author thinks it is impossible to distinguish during the attack these three different kinds of pain: First, a more or less continuous throbbing pain; second, a sharp, lightning-like pain, succeeded frequently by a burning or tingling sensation; third, a superficial cutaneous hyperæsthesia, the "points douloureux" of Valleix. The throbbing pain he attributes to a delitation of the vessels of the nerves themselves; the sharp, lightning-like pain to a more acute compression of the nerve, brought about by a sudden alteration in the blood pressure, or an increased delitation of the already dilated blood-vessels, perhaps from such slight causes that they usually escape notice. With regard to the production of the tender spots of Valleix, the explanation is ingenious. As the nerve, he says, passes on its course, it gives off filaments to the surrounding parts, and these filaments come off from the main trunk in a definite order from without inwards; therefore the most superficial filaments of a nerve-trunk are those which supply the tissues in the immediate vicinity. But it is these very filaments which suffer most when a nerve is compressed in the osseous or other canal; and therefore, by the law of eccentric projection, there is a hyperæsthesia in the part supplied by those filaments—in fact, a tender spot. And since these filaments suffer most, it is not at all unlikely that, as the attack progresses, the prolonged pressure would temporarily destroy their function and cause local anæsthesia; and this is exactly what occurs—hyperæsthesia being more likely to occur at the earlier part of an attack, and anæsthesia later.

He considers the treatment of neuralgia under three headings, namely: 1, external local; 2, external remote; 3, internal.

1. Of the local methods of treatment, such operative methods as incision and division of the nerve sufficiently explain themselves. Stretching the nerve and acupuncture seem to act in the same way, probably by causing a solution of continuity in the sheath, and thus relieving tension, and deminishing the swelling of the nerve. As regards stretching however, Callender considers that "it is probable that the stretching is of use by numbing the nerve for a short time." If this be really true, the natural explanation would be that the sensory irritation resulting from the hyperæmia would for a time cease, and on its cessation the reflex dilatation of the blood-vessels would also be brought to an end. Electricity, again, in common with belladonna and heat or cold locally applied would contract blood-vessels. Belladonna, volatile oils and their stearoptenes, [1] such as thymol and menthol, also chloroform, not only contract the blood-vessels, but also diminish the sensibility of the part, while aconite locally applied acts purely as an anodyne.

2. Of the external and remote methods of treatment, counter-irritation over the upper part of the dorsal spine, which apparently does good in some cases, must be mentioned, though perhaps somewhat difficult to explain, unless possibly it has some action on the sympathetic vaso-motor fibres which emerge from the cord about this level in the anterior roots of the lower cervi cal and upper dorsal nerves (Foster). Ligature of the carotid is an eminently successful, though somewhat heroic treatment, and has been very successfully employed by Dr. Patruban in many cases of severe neuralgia—this, of course, acting directly on the blood-vessels, the idea of the operation having originated from the relief obtained in some cases by simple pressure on the carotid.

3. Of the internal treatment of neuralgia the substances used fall naturally into four groups:

(1.) Those which contract blood-vessels, such as strychina, atropia, and its allies, bromide of camphor, digitalis, ergot, volatile oils, such as turpentine, chloride of ammonium and quinine, though certain of these also have a specific action on sensory nerves in addition.

(2.) A second group consists of bodies which act as general tonics, such as quinine, iron, strychnia, phosphorus and arsenic. The experience, however, of arsenic and phosphorus seems to indicate that they have some more direct action than simple tonics.

(3.) A third group consists of simple anodynes or sedatives,

1 Solid crystalline compounds seperable from volatile oils by cold.

such as bromide of potasium. This, however, like chloral, diminishes the pulse tension, and in addition diminishes reflex excitability by depression of the peripheral sensory filaments. Other drugs, such as cannibis indica and morphia, seem to act as simple anodynes.

(4.) The fourth group consists of the usual tell-tale drugs, which are always appearing, and of the action of which no very apparent explanation can be given. Amongst these gelsemium sempervirens is a prominent member, and probably also arsenic and phosphorus.

He concludes by saying that there are, then, symptomatic, pathological, etiological, and therapeutic reasons for believing that the blood vessels are at fault in cases of neuralgia, and therefore, on this view, it seems necessary to consider true neuralgia as a sympathetic neurosis, affecting certain tracts of cerebro-spinal nerves, resulting in simple dilatation of the vessels of these nerves, brought about by unknown causes, or by reflex irritation, or possibly by the specific action of certain poisons in the blood.

